# Challenges to Licensure of Enterovirus 71 Vaccines

**DOI:** 10.1371/journal.pntd.0001737

**Published:** 2012-08-28

**Authors:** Min-Shi Lee, Fan-Chen Tseng, Jen-Ren Wang, Chia-Yu Chi, Pele Chong, Ih-Jen Su

**Affiliations:** 1 National Institute of Infectious Diseases and Vaccinology, National Health Research Institutes, Taiwan; 2 Institute of Basic Medical Sciences, National Cheng Kung University Medical College, Tainan, Taiwan; 3 Department of Medical Laboratory Science and Biotechnology, National Cheng Kung University, Tainan, Taiwan; The George Washington University Medical Center, United States of America

## Abstract

Human enteroviruses usually cause self-limited infections except polioviruses and enterovirus 71 (EV71), which frequently involve neurological complications. EV71 vaccines are being evaluated in humans. However, several challenges to licensure of EV71 vaccines need to be addressed. Firstly, EV71 and coxsackievirus A (CA) are frequently found to co-circulate and cause hand-foot-mouth disease (HFMD). A polyvalent vaccine that can provide protection against EV71 and prevalent CA are desirable. Secondly, infants are the target population of HFMD vaccines and it would need multi-national efficacy trials to prove clinical protection and speed up the licensure and usage of HFMD vaccines in children. An international network for enterovirus surveillance and clinical trials is urgently needed. Thirdly, EV71 is found to evolve quickly in the past 15 years. Prospective cohort studies are warranted to clarify clinical and epidemiological significances of the antigenic and genetic variations between different EV71 genogroups, which is critical for vaccine design.

Enteroviruses (EVs) are single-stranded, positive-sense RNA viruses in the Picornaviridae family. They cause various clinical manifestations, including cutaneous, visceral, and neurological diseases [1]. There are more than 100 human enterovirus serotypes, including 3 poliovirus serotypes, 23 coxsackievirus A (CA) serotypes, 6 coxsackievirus B (CB) serotypes, 31 echovirus serotypes, and 39 numbered enterovirus serotypes (EV68-71, EV73-102, EV104-107, and EV109). Human enteroviruses can be phylogenetically classified into four species (A, B, C, and D) [1], [2]. For many years polioviruses were the most important enteroviruses since they caused large outbreaks of paralytic disease before poliovirus vaccines were available. Although EV71 was first isolated in 1969, a retrospective analysis shows that this virus circulated in the Netherlands as early as 1963. Recent molecular evolution studies predicted that EV71 could emerge in the human population around 1941 [3], [4]. Recently, EV71 repeatedly caused life-threatening outbreaks of hand-foot-mouth disease (HFMD) with neurological complications in Asian children. The neurological manifestations progress very quickly and range from aseptic meningitis to acute flaccid paralysis and brainstem encephalitis [2]. Due to its tremendous impact on healthcare systems, development of EV71 vaccines is a national priority in some Asian countries. Vaccine candidates are being evaluated in humans. Several review articles related to EV71 epidemiology, clinical management, pathogenesis, and preclinical vaccine development have been published [1], [2], [5]–[7]. In this review we focus on disease burden of enteroviruses and EV71 in Asian countries, importance of the antigenic and genetic variations, and the potential challenges to licensure and delivery of EV71 vaccines.

## Disease Burden of Enteroviruses

HFMD is known as a common pediatric illness. Coxsackievirus A16 (CA16) was the first viral agent isolated from patients with HFMD [8]–[10]. Later CA4, CA5, CA6, CA9, and CA10 as well as Coxsackievirus B (CB) were also found as etiologic agents for HFMD [11]–[16]. In the Asia Pacific region, a series of HFMD epidemics with neurological complications and severe outcomes in young children had occurred since late 1990s, which prompted the establishment of national surveillance of HFMD and/or enteroviruses in some Asian countries. Japan was the first country in Asia to establish a comprehensive EV surveillance with symptom-reporting and virus identification using molecular typing (http://idsc.nih.go.jp/idwr/ydata/report-E.html). Table 1 show the top 5 EV serotypes isolated in Japan during 2000–2009, and different serotypes predominated each year (http://idsc.nih.go.jp/iasr/virus/virus-e.html). While EV71 seemed to have an epidemic every 3 years, CA16 was more often the prevalent type. Infections by HEV-A can also be presented with herpangina, which can sometimes be difficult to differentiate from HFMD. Annual cases of HFMD and herpangina reported from sentinel clinics ranged between 145,000 and 352,000.

**Table 1:** Top 5 serotypes of enterovirus isolated in Japan, 2000–2009.

Rank	Laboratory Confirmed Serotypes (%)									
	2000 (*N* = 3,229)	2001 (*N* = 2,341)	2002 (*N* = 4,561)	2003 (*N* = 3,490)	2004 (*N* = 2,310)	2005 (*N* = 1,937)	2006 (*N* = 2,571)	2007 (*N* = 2,194)	2008 (*N* = 2,338)	2009 (*N* = 1,865)
**1**	EV71 (15.2)	E11 (13.9)	E13 (46.2)	EV71 (18.9)	CA4 (16.8)	CA6 (21.2)	E18 (22.3)	CB5 (19.0)	CA16 (20.2)	CB3 (12.3)
**2**	CB5 (9.1)	CA16 (13.7)	CA16 (9.3)	E6 (14.3)	CB1 (9.5)	CA16 (13.9)	EV71 (12.5)	CA16 (15.8)	E30 (10.2)	CA9 (12.1)
**3**	CA10 (8.8)	CB5 (9.3)	E11 (8.5)	E30 (14.1)	E6 (9.3)	CB3 (12.2)	CA4 (12.3)	CA6 (11.7)	CA4 (8.1)	CA6 (10.1)
**4**	E9 (7.7)	CA2 (6.9)	CA4 (6.0)	CA10 (11.0)	CA16 (8.2)	CA9 (5.3)	CA16 (7.6)	E30 (10.1)	CB5 (7.8)	CA10 (8.4)
**5**	E25 (7.5)	CA4 (6.1)	CB2 (5.9)	CA4 (4.1)	CA2 (7.7)	E9 (5.2)	CA9 (6.4)	EV71 (6.3)	CA2 (6.4)	EV71 (4.7)

Data source: http://idsc.nih.go.jp/iasr/index.html.

Taiwan performed both disease-reporting, systematic sampling of clinical specimens for virus isolation for the EV surveillance since 1999 [17]. Case numbers of HFMD and herpangina reported from sentinel clinics ranged between 93,000 and 140,000 annually. Table 2 shows the top 5 EV serotypes isolated each year during 2000–2009, but the ranking was more reliable after 2003, when molecular typing was employed. EV71 and CA16 were the predominant serotypes in 2000–2003, and rebound in 2004–2005 and 2006–2008. Among cases with EV71 or CA16, HFMD and herpangina were recorded in 21% and 42.7%, respectively [17].

**Table 2:** Top 5 serotypes of enterovirus isolated in Taiwan, 2000–2009.

Rank	Laboratory Confirmed Serotypes (%)									
	2000 (*N* = 1,310)	2001 (*N* = 2,114)	2002 (*N* = 1,839)	2003 (*N* = 2,066)	2004 (*N* = 2,389)	2005 (*N* = 2,518)	2006 (*N* = 2,801)	2007 (*N* = 1,959)	2008 (*N* = 3,807)	2009 (*N* = 1,666)
**1**	EV71 (26.3)	E30 (21.5)	CA16 (16.2)	CA16 (35.8)	CA4 (23.8)	CB3 (31.6)	CA4 (27.0)	CA16 (30.4)	CA2 (32.1)	CA6 (31.1)
**2**	CA16 (18.7)	EV71 (20.8)	E6 (12.6)	E9 (7.5)	CA10 (22.7)	CA16 (25.4)	CA2 (15.6)	CA6 (24.3)	EV71 (25.7)	CA10 (27.8)
**3**	E9 (10.5)	CA16 (15.6)	EV71 (10.1)	EV71 (7.0)	CB4 (16.5)	EV71 (15.8)	CA5 (12.0)	CA10 (22.0)	CB4 (10.3)	CA4 (13.8)
**4**	CB3 (7.5)	E6 (10.4)	CB5 (9.4)	E11 (6.6)	EV71 (9.5)	CA6 (6.6)	E18 (9.8)	CA4 (5.8)	CB1 (3.9)	CB1 (5.5)
**5**	CB4 (4.5)	CB4 (2.3)	CA4 (6.0)	CA2 (6.5)	CA6 (2.6)	CA5 (5.9)	CB2 (7.4)	E6 (4.1)	CA16 (3.8)	CA5 (5.3)

Data source: http://www.cdc.gov.tw.

EV71 surveillance in Malaysia was through sentinel clinics and virological identification in some parts of the country since 1997, and virological identification for CA16 was added after 2000 (http://sydney.edu.au/medicine/apnet/). Virological monitoring in Sarawak indicated that EV71 epidemics occurred every 3 years in 1997, 2000, and so on, but CA16 can be detected in both epidemic and inter-epidemic periods [18]. However, the cyclical pattern was not observed in Kuala Lumpur [19]. Reports of other HEV-A serotypes had not been seen, probably because these types were not included in the surveillance.

In Singapore, cases of HFMD were notified to the Ministry of Health, and clinical specimens were sampled randomly for EV identification. During 2001–2007, HFMD epidemics were detected in 2002 and 2005–2007, with more than 15,000 cases reported each year [20]. Major EV serotypes isolated from HFMD cases were CA16 and EV71, and CA6 was also an important etiology in 2006 (Table 3). Overall, in 7 years, CA16 and EV71 were detected in 40% and 30% of the cases, respectively [20]. Then, in the 2008 HFMD outbreak, the largest one in the decade, nearly 30,000 cases were reported, with CA6 and EV71 being predominant strains, followed by CA10 [21].

**Table 3:** Top serotypes of enterovirus isolated from HFMD patients in Singapore, 2001–2007.

Rank	Laboratory Confirmed Serotypes (%)						
	2001 (*N* = 178)	2002 (*N* = 210)	2003 (*N* = 50)	2004 (*N* = 21)	2005 (*N* = 76)	2006 (*N* = 145)	2007 (*N* = 94)
**1**	EV71 (45.6)	CA16 (76.2)	EV71 (68.0)	CA16 (66.8)	EV71 (52.7)	EV71 (45.5)	CA16 (64.9)
**2**	CA6 (18.5)	CA6 (11.9)	CA10 (16.0)	CA2 (14.3)	CA16 (43.4)	CA6 (35.8)	CA6 (10.6)
**3**	CA16 (15.7)	CA10 (6.7)	CA16 (10.0)	CA10 (9.5)	CA10 (2.6)	CA16 (5.5)	CA10 (9.6)
**4**	CA4 (10.7)	EV71 (3.8)	CA4 (6.0)	CA4 & EV71 (4.7)	Echo (1.3)	CA2 (3.5)	EV71 (8.5)

In China, HFMD had been reported as early as the 1980s, but its epidemic and clinical problems were not well recognized until 2007. From the record of China Ministry of Health (MOH), the first epidemic occurred in 2007 with more than 80,000 cases reported, including 17 deaths (http://www.chinacdc.cn/). Virological identification was only performed in a few hospitalized cases, and the majority was detected with EV71. In 2008, the HFMD epidemics spread to multiple provinces, resulting in nearly 0.5 million reported cases and 126 deaths, and in May 2008, China MOH listed HFMD as a notifiable disease (http://www.moh.gov.cn/publicfiles/). In 2009, more than 1.1 million cases and 353 deaths were reported. Epidemics continued to escalate in 2010, with 1.8 million cases reported and 905 deaths, and it slowly alleviated in the first half of 2011, with 0.7 million cases and 230 deaths.

Overall, it is hard to compare the absolute case numbers of HFMD reported in different countries with different case definitions, data collections, and laboratory procedures. However, EV71 and CA are frequently found to co-circulate and cause HFMD in some Asian countries. Therefore, an international network of enterovirus surveillance systems is urgently needed to understand disease burden of enteroviruses; in particular, an Asian working group could be formed under the Asia-Pacific Economic Cooperation (APEC) platform.

## Clinical Spectrum and Disease Burden of EV71

According to previous clinical studies conducted in northern Taiwan, symptomatic EV71 infections can progress through four stages: HFMD/herpangina (Stage 1), CNS involvement (Stage 2), cardiopulmonary failure (Stage 3), and convalescence (Stage 4) [2]. Recent follow-up studies further demonstrated that EV71 infection could cause long-term sequelaes including neurological development and cognitive function [22]. In a prospective hospital-based case-finding study, 21% of 183 EV71 infections in children <18 months of age developed neurological complications such as meningitis and encephalitis [23]. Based on national severe enterovirus surveillance and two cross-section serological surveys, Lu et al. estimated that 130,617 Taiwanese children aged <3 years were infected with EV71 infections in 1998 and that 273 (0.21%) of these infected children developed neurological complications [24].Overall, the prospective hospital-based case-finding study would overestimate the proportion of EV71 infections with neurological complications and the national surveillance data would underestimate the proportion of EV71 infections with neurological complications (Table 4).

**Table 4:** Comparisons on clinical spectrum of enterovirus 71 infections in children.

Reference	Lee et al. [28] (*N* = 28)	Chang et al. [27] (*N* = 172)	Lu et al. [24]	Chang et al. [23] (*N* = 183)
Study design	Prospective cohort	Retrospective cross-sectional serosurvey	Retrospective cross-sectional serosurvey	Prospective hospital-based case finding
Laboratory diagnosis	Neutralizing antibody seroconversion	Neutralizing antibody seroprevalence	Neutralizing antibody seroprevalence	Virus isolation, serum IgM test, neutralizing antibody ≥4-fold rise
Year of epidemic (EV71 Genotype)	2008–09 (B5)	1998 (C2)	1998 (C2)	2001–02 (B4)
Age (years)	<3	<3	<3	<19
Asymptomatic infection	29%	No data*	No data	6%
Non-specific illnesses	32%	No data*	No data	13%
HFMD/Herpangina	39%	37%	No data	60%
Neurological complications	No data	<0.6%	0.21%	21%

HFMD, hand-foot-mouth disease.

* This study did not collect data about mild illness, but it indicates that 63% of infections were asymptomatic or caused non-specific illness.

EV71 was first isolated in California (United States of America) in 1969. Since then, EV71 has been detected globally [2], [3], [5], [25]. Globally, two patterns of the EV71 epidemic have been reported: small-scale outbreaks with little CNS-complicated cases and deaths, and large-scale outbreaks with frequent CNS-complicated cases and deaths. The latter pattern occurred in Bulgaria in 1975, in Hungary in 1978, in Malaysia in 1997, in Taiwan in 1998, in Singapore in 2000, in southern Vietnam in 2005 and 2007–2009, in Brunei in 2006, in Korea in 2009, and in China in 2007–2009 (Table 5) [2], [26]. Since EV71 mortality rates are heavily affected by healthcare accessibility and standards, a more relevant clinical definition, such as CNS complication, should be also used to quantify disease burden of EV71 infections in prevalent areas.

**Table 5:** EV71-related severe and fatal cases in Asia, 1997–2009.

Country	Year	Severe Case No.*	Death No.
Taiwan	1998	405	78
	2000	152	25
	2001	187	27
	2002	55	7
	2003	44	4
	2004	20	5
	2005	82	7
	2008	346	14
	2009	25	2
Malaysia	1997	No data	29
	2000–03	185	4
	2006	436	6
Singapore	2000	No data	5
	2001	No data	3
Vietnam (southern part)	2005	51	3
	2007	No data	23
	2008	No data	25
	2009	No data	23
Brunei	2006	No data	3
Korea	2009	92	2
China	2007	No data	>27
	2008	1,165	126
	2009	10,509	353

* Different countries may have different definitions and surveillance systems, so these data should be interpreted with caution.

To design clinical trials of EV71 vaccines, age-specific incidence rates of EV71 infections are required to identify target populations, estimate disease burdens, define endpoints of clinical efficacy, and calculate the sample size for efficacy trials. Taiwan has a national surveillance system for severe enterovirus infections since 1998. Age-specific incidence rates of EV71-related severe infections during the 1998 epidemic have been estimated to be 27.3, 37.1, 30.0, and 23.1 per 100,000 for children aged <6, 6–11, 12–23, and 24–35 months, respectively, which would be too low to be selected as a suitable clinical endpoint [27]. Alternatively, EV71-related mild illness such as herpangina and HFMD could be suitable clinical endpoints. An infant prospective cohort study initiated in 2006 in northern Taiwan and a nationwide EV71 epidemic occurred in 2008–2009; the age-specific incidence rates of EV71 infection during the 2008–2009 epidemic were observed to be increased from 1.71 per 100 person-years at 0–6 months of age infants to 4.09, 5.74, and 4.97 per 100 person-years at 7–12, 13–24, and 25–36 months of age children groups, respectively. In addition, the cumulative incidence rate was 15% by 36 months of age, 39% of EV71 infections developed HFMD/herpangina, and 29% of EV71 infections were asymptomatic in young children [28]. A retrospective serosurvey also found that 37% of seropositive children reported to develop HFMD/herpangina during the 1998 nationwide epidemic in Taiwan (Table 4) [27]. Overall, development of EV71 vaccines should target children <6 months of age, and EV71-related mild illness such as herpangina and HFMD could be used as the clinical endpoints of efficacy trials. To speed up the licensure of EV71 vaccines in young infants, a multination randomized controlled efficacy trial is necessary to prove the clinical protection in this age group. Asian countries do not have harmonized enterovirus surveillance systems at the moment, and an international network for enterovirus surveillance and clinical trials is urgently needed and could be organized under the APEC public health network.

## Genetic and Antigenic Variations

Based on serum neutralization tests using hyperimmune animal antisera, EV71 is classified as a single serotype. According to analysis of VP1 sequences, however, EV71 was phylogenetically divided into three distinct genogroup: A, B, and C [1], [2]. Genogroup A composes the EV71 strain (BrCr-CA-70), which was identified in 1970 in the United States but had not been detected afterwards until 2008. In an investigation of HFMD outbreak in central China in 2008, Yu et al. identified 5 EV71 isolates that were closely related to genotype A based on analysis of VP1 genes [29]. Reasons of the reemergence of genotype A in central China is not clear, and the full genomic sequences of the isolates should be performed to clarify the issue.

In contrast, genotype B and genotype C continued to circulate around the world after the 1970s and the 1980s, respectively. Genotypes B and C can be further divided into 5 genotypes, respectively: B1 to B5 and C1 to C5. Recently, retrospective studies show that a genotype B0 virus circulated in the Netherlands as early as 1963 and a genotype C0 virus circulated in Japan as early as 1978 [3], [25]. Among these genotypes, genotype C3 was only seen in Korea in 2000, and others were found to spread either globally or regionally (Table 6) [30]. In the 1970s, only genotypes B1 and B2 have been reported to play roles in HFMD epidemics in America and Europe [30]. In the 1980s, genotype B2 became the predominant genotype and this EV71 lineage continued circulating in Japan, Taiwan, the United States, the Netherlands, and Australia [30]. However, a new genotype C1 appeared and replaced the predominant genotype B2 and became the major genotype in the late 1980s and the early 1990s (Table 6).

**Table 6:** Distribution of EV71 genotypes throughout the world from 1997 to 2010.

	1997	1998	1999	2000	2001	2002	2003	2004	2005	2006	2007	2008	2009	2010
Malaysia	C1,C2, **B3**,B4	C1	C1	**B4**,C1		C1	**B5**,C1		B5,C1	B5				
Singapore	B3,B4	B3,C1	B3	**B4**	B4	B4,C1				**B5**		**B5**		
Taiwan		B4,**C2**,C4	B4	**B4**	**B4**	B4,C4	B4,B5	**C4**	**C4**	C5	B5,C5	B5	**B5**	C4
Japan	**C2**, B3,B4	C2	C2	C2,**B4**	C2	B4,C2,C4	**C4**,**B5**	C4		C4	C4			
China		C4		C4	C4	C4	C4	C4			C4	A,**C4**	C4	
Vietnam									C1,C4,**C5**					
Australia		C2	**B3**,C2	B4,C1	B4,C1	C1	C1	C4						
Korea				C3									C4	
The Netherlands	C1,C2		C2	C2	C1	C1,C2		C1,C2	C1,C2		C1,**C2**	C2		
United Kingdom		C1	C1,C2	C1	C1	C1		C1		C1,C2				
Norway						C1	C1							
Austria					C1	C1	C1	C4						

Bold indicates predominant genotype.

Since 1997, the largest wave of EV71 epidemics in the history has killed thousands of children around the Asia-Pacific region. In the first HFMD epidemic in the Asian-Pacific region, various genotypes including genotypes C1, C2, B3, and B4 were observed in Malaysia in 1997, and genotype B3 was the predominant strain in the outbreak. In 2000 and 2003, genotype B4 and genotype B5 replaced genotype B3 and became the predominant genotypes in the later outbreaks, respectively, according to the result of sentinel surveillance from 1998 to 2005 in Sarawak, Malaysia. Genotype C1 appeared sporadically between the three outbreaks in 1997, 2000, and 2003. In short, intra-genogroup B shifts were observed from the late 1990s to early 2000s in Malaysia, including genotypes B3 and B4 (during 1997 to 2000) and B4 to B5 (during 2000 to 2003), and genotype C1 is the only genotype that had continually circulated in Sarawak (Table 6) [30].

In the same period, two outbreaks in 2000 and 2006 were detected in Singapore. After the 1997 epidemic in Malaysia, only three genotypes (B3, B4, and C1) were detected from 1997 to 1999 in Singapore. Afterward, the first large outbreak caused by genotype B4 in 2000 was reported in Singapore. Genotypes B4 and C1 co-circulated after the 2000 epidemic and an intra-genogroup B shift (B4 to B5) appeared and this genotype B5 replacement resulted in an outbreak in 2006 (Table 6) [30].

Instead of intra-genogroup B shift, inter-genogroup shifts between B and C have been detected in Taiwan. In 1998, Taiwan observed a large HFMD outbreak with 129,106 severe cases and 78 fatal cases [31]. A genetic analysis shows that genotype C2 was the predominant strain, but approximately 10% of EV71 isolates in the same period belonged to genotype B4. Although the number of cases of enterovirus infections dramatically decreased in Taiwan in 1999, another large HFMD outbreak occurred in 2000 and 2001. A genotype shift from C2 to B4 was observed. Afterwards, genotype B4 circulated in Taiwan until 2004 and genotype C4 replaced B4 as the predominant strain in 2004 and 2005. In contrast to the low EV71 activity between 2006 and 2007, genotype B5 activity significantly increased in 2008–2009 and dozens of fatal cases with neurological diseases were reported. After the 2008–2009 epidemic, EV71 returned to low activity in Taiwan [30]. In the other countries of the Asia-Pacific region, EV71 outbreak did not show a regular pattern similar to the countries mentioned above. In China, only a single genotype C4 (which contains two lineages, C4a and C4b) continually circulated and resulted in the HFMD outbreak in 2007–2009 [32]–[34]. In Vietnam, genotypes C1, C4, and C5 co-circulated and C5 was the predominant one in the HFMD outbreak in 2005 [35]. From an evolution prospective, a recent analysis of 628 EV71 VP1 sequences showed that EV71 may have emerged in the human population around 1941, and rises in genetic diversity are correlated with the onset of epidemics, driven in part by the emergence of novel EV71 genotypes [4]. However, recombination might be the likely mechanism for the emergence of new enterovirus serotypes [30]. Overall, the mechanism of the genogroup shift and gene recombination is not clear and needs to be clarified using a longitudinal cohort study and full genome analysis.

Does the observed genetic variations cause antigenic variations? This is an important question related to the prediction of disease outbreaks and the selection of vaccine strains. It seems that EV 71 has mutated more quickly in the last 15 years and more genotypes are now spreading globally. Kung et al. did not detect significant antigenic differences between genotypes B4 and C4 viruses using acute-phase sera from hospitalized EV71 patients [36]. A serological survey in healthy Japanese children and adults detected partial antigenic differences between genotype B5 and A viruses but not among genogroup B and C viruses that were previously circulating in Japan [37]. Using sera collected from young children with primary infection of genotype B5, two studies detected partial antigenic differences between genogroup B and C but not between viruses in the same genogroup (B5 and B4 viruses) [38], [39]. By constructing an antigenic map, however, Huang et al. detected antigenic differences between genogroups B and C and also between B5 and B4 viruses [40]. It is hard to compare different studies that employed different human sera and laboratory procedures, in particular the cell lines used in the neutralization assay. A network to harmonize laboratory procedures including standard sera and viruses is required to make the comparison possible. Moreover, the clinical and epidemiological significance of the antigenic variation requires longitudinal serological studies to clarify.

In the 1970s, a novel enterovirus causing acute hemorrhagic conjunctivitis was identified right before the identification of EV71 and named as EV70. EV70 had caused large-scale epidemics in Africa and Asia in the 1970s. Since then, only local outbreaks were reported [41]. Interestingly, EV70 and EV71 had a similar pattern in the 1970s and 1980s, but only EV71 has continuously caused large-scale epidemics in Asia-Pacific countries since 1997. It would be worthy to elucidate the evolutionary mechanisms and epidemiological patterns of EV70 and EV71.

## Clinical Development of EV71 Vaccines

The success of live-attenuated and inactivated poliovirus vaccines (IPV) in preventing poliomyelitis indicates the potential for preventing EV71 by vaccination. After the Bulgaria epidemic in 1975, an inactivated EV71 whole virus vaccine candidate was produced in Moscow using the similar manufacturing process of IPV and was evaluated in Bulgaria in 1976. This EV71 vaccine candidate was well tolerated and immunogenic in children 1–4 years of age [42]. For the practical reason of having no further outbreaks of EV71, the Bulgaria vaccine candidate was not further evaluated for its clinical efficacy, and no potency assay to quantify vaccine antigens had been developed. With these backgrounds, the inactivated EV71 vaccine is the candidate being evaluated in clinical trials in China, Singapore, and Taiwan (Table 7) [43]. All vaccine candidates are inactivated whole virus particles, but different cell lines and virus genotypes are used in different countries (C4 in China, B in Singapore, and B4 in Taiwan). It would be important to compare productivity of different cell lines and virus strains and evaluate vaccine-induced cross-reactive neutralization antibody titers against all genogroups in naïve populations.

**Table 7:** EV71 vaccine candidates in clinical trials.

Organization (Country)	Cell Line	Formulation (Virus Genotype)	Reference
Beijing Vigoo; CNBG (China)	Vero cell	Inactivated virus (C4)	www.clinicaltrials.gov; www.cnbg.com.cn
Sinovac (China)	Vero cell	Inactivated virus (C4)	www.sinovac.com; www.clinicaltrials.gov
PUMC (China)	KMB-17 cell	Inactivated virus (C4)	www.pumc.edu.cn
National Health Research institutes (Taiwan)	Vero cell	Inactivated virus (B4)	www.nhri.org.tw; www.clinicaltrials.gov
Inviragen (Singapore)	Vero cell	Inactivated virus (B)	www.inviragen.com

CNBG, China National Biotech Group; PUMC, Peking Union Medical College.

Other major difference in these vaccine candidates are the potency assay used for product release and quantification of vaccine antigens. At the moment, the standard reagents for potency assay of EV71 vaccines are not available. Based on experiences learned from IPV vaccine, several antigenic formats exist during production of IPV and these different antigenic formats showed various capacities for inducing neutralizing antibody responses. Therefore, under the coordination of World Health Organization (WHO), reference reagents were established based on one specific antigen and used establishing assays to ensure the consistent potency of IPV. Moreover, international collaborations on harmonization of potency assays of IPV are critical for the increasing supply of IPV produced by different manufacturers [44]. Therefore, regulatory authorities in Asian countries where EV71 vaccine development programs exist would benefit from having an international network to establish international reference reagent for use in potency assays of EV71 vaccines.

## Conclusions

EV71 is highly contagious and causes life-threatening outbreaks in children in tropical Asia. Several EV71 vaccine candidates are being evaluated in clinical trials in this region. To speed up the licensure of EV71 vaccines in epidemic countries, the following challenges should be seriously addressed. Firstly, EV71 and CA are frequently found to co-circulate and cause similar clinical symptoms, such as HFMD and herpangina. The indistinguishable clinical presentations of EV71 and CA may reduce public confidence and acceptance of EV71 vaccines. A combined polyvalent vaccine that can provide protection against EV71 and other prevalent CA are therefore desirable commercially and administratively. Secondly, infants are the target population of EV71 vaccines and it would need multi-nation randomized controlled efficacy trials to prove clinical protection in this age group and justify the licensure and usage of EV71 vaccines in children. An international network for enterovirus surveillance and clinical trials is urgently needed to help design and conduct efficacy trials in epidemic countries. Thirdly, EV71 and CA are found to recombine and tend to evolve quickly in the past 15 years. Prospective cohort studies are warranted to clarify clinical and epidemiological significances of the antigenic and genetic variations, which are critical to selection of vaccine strains. Last but not least, regulatory authorities in Asian countries where EV71 vaccine development programs exist would benefit from having an international network to establish international reference reagents (standardized cell lines, virus seed, and antisera) for use in potency assays of EV71 vaccines.

**Key Learning Points**

- EV71 and CVA are frequently found to co-circulate and cause similar clinical symptoms, such as HFMD and herpangina. The indistinguishable clinical presentations of EV71 and CVA may reduce public confidence and acceptance of EV71 vaccines.
- A combined polyvalent vaccine which can provide protection against EV71 and other prevalent CVA are therefore desirable commercially and administratively.
- Infants are the target population of HFMD vaccines and it would need multi-national randomized controlled efficacy trials to prove clinical protection in this age group and justify the licensure and usage of HFMD vaccines in children. An international network for enterovirus surveillance and clinical trials is urgently needed.
- EV71 and CVA are found to recombine and tend to evolve quickly in the past 15 years. Prospective cohort studies are warranted to clarify clinical and epidemiological significances of the antigenic and genetic variations, which are critical to vaccine design.
- Regulatory authorities in Asian countries where EV71 vaccines development programs exist would be benefited from having an international network to establish international reference reagents for use in potency assays of EV71 vaccines.

**Top 5 Papers**

1. Solomon T, Lewthwaite P, Perera D, Cardosa MJ, McMinn P, et al. (2010) Virology, epidemiology, pathogenesis, and control of enterovirus 71. Lancet Infect Dis 10: 778–790.
2. Lee MS, Chang LY (2010) Development of enterovirus 71 vaccines. Expert Rev Vaccines 9: 149–156.
3. Tee KK, Lam TT, Chan YF, Bible JM, Kamarulzaman A, et al. (2010) Evolutionary genetics of human enterovirus 71: origin, population dynamics, natural selection, and seasonal periodicity of the VP1 gene. J Virol 84: 3339–3350.
4. Chang LY, Huang LM, Gau SS, Wu YY, Hsia SH, et al. (2007) Neurodevelopment and cognition in children after enterovirus 71 infection. N Engl J Med 356: 1226–1234.
5. Shindarov L, Drozdov S, Grachev V, Umansky K, Nikolaevsky G, et al. A study on anti-enterovirus 71 vaccine; 2–6 October 1978; Verna, Bulgaria. pp. 385–388.
